# Barriers and Facilitators to Medicines Use in Patients With Vision Impairment: A Theory‐Informed Qualitative Study of Patients and Caregivers

**DOI:** 10.1111/hex.70234

**Published:** 2025-03-21

**Authors:** Basma Y. Kentab, Heather E. Barry, Sinaa A. Al‐Aqeel, Carmel M. Hughes

**Affiliations:** ^1^ Department of Clinical Pharmacy, College of Pharmacy King Saud University Riyadh Saudi Arabia; ^2^ Primary Care Research Group, School of Pharmacy Queen's University Belfast Belfast Northern Ireland UK

**Keywords:** caregivers, medication use, patients, qualitative, Theoretical Domains Framework, vision impairment

## Abstract

**Introduction:**

Previous studies have highlighted numerous challenges with medicines use for patients with vision impairment, but evidence is lacking on interventions to support safe and effective medicine use for this population. This study aimed to identify potential barriers/facilitators of medicines use from the viewpoint of patients/caregivers, to establish a theory‐informed foundation for a future intervention.

**Methods:**

Semi‐structured interviews were conducted with patients with vision impairment and their caregivers in Saudi Arabia. Participants were recruited from low‐vision clinics and a non‐profit organisation. The Theoretical Domains Framework (TDF) informed the topic guide and served as the theoretical basis for examining participants' behaviours. Interviews explored the barriers/facilitators to obtaining and taking medicines (i.e. the target behaviours). Data were analysed using the framework method and content analysis. Key TDF domains were identified by assessing the relative frequency of themes, the existence of conflicting themes and the perceived theme impact on target behaviours.

**Results:**

Twelve patient/caregiver dyads and 18 individual patients were interviewed. Patients' ages ranged from 19 to 88 years, with 21 females and 9 males. Patients/caregivers demonstrated good knowledge of medicines used, and resourcefulness in finding methods to manage medication use (Domains: ‘Knowledge’ and ‘Skills’). ‘Environmental context and resources (ECR)’ and ‘Social influences’ were the two most coded domains. Participants reported the usefulness of several resources including pill organisers and smartphones but described problems with pharmacy environments (Domain: ‘ECR’). Caregivers played a major role in assisting patients with medicines. Participants had some positive encounters with pharmacists but reported difficulties in discussing medication issues with them. Many participants had a narrow view of the pharmacist's role (Domain: ‘Social influences’). Maintaining a routine was a major facilitator under the ‘Memory, attention and decision processes’ and ‘Behavioural regulation’ domains. Six TDF domains were considered ‘key’ to participants' behaviours.

**Conclusions:**

This study is the first to utilise a theoretical approach to understand the behaviour of patients with vision impairment in relation to medication use. It provides a comprehensive understanding of the role of caregivers and what influences their own behaviours. Findings will inform the future development of an intervention to support safe and effective medicine use for patients with vision impairment.

**Patient or Public Contribution:**

An author met with an administrator at the ‘National Association of the Blind’ (Kafeef) in Riyadh to enhance the research team's understanding of vision impairment, and the practicality and logistics for identifying and recruiting patients. A draft of the interview guides was piloted with two patients and their caregivers and they were asked for feedback on the questions with amendments made accordingly.

## 1 Introduction

Understanding the experiences of patients with vision impairment and the challenges they encounter when using medicines is paramount to tailoring interventions to best fit the needs of this population. Challenges with medicine use in patients with vision impairment include differentiating medications, identifying doses and expiry dates, and medication adherence [1, 2, 3]. Patients have also been found to be more susceptible to medication errors [4]. These data originate from quantitative research that typically used surveys to describe the medicines‐related experiences of this population rather than what affects their behaviours. Very few studies have employed a qualitative approach to gain an in‐depth understanding of the factors that influence medicine use behaviour in patients with vision impairment and their caregivers. In three studies that utilised an interview‐based approach, issues highlighted difficulties with differentiating between medications, reading and/or remembering instructions and expiry dates, measuring liquid medication doses, physically navigating pharmacies and communicating with pharmacy personnel [5, 6, 7, 8]. In their scoping review of the pharmaceutical needs of people with sensory loss, Killick et al. recommended that future research in this area should explore the needs of patients with vision impairment in more detail, consider the role of caregivers in facilitating medicines use, and involve patients in suggesting possible improvements to care provided by pharmacists [9].

A systematic review identified a lack of pharmacists' interventions to support safe and effective medicine use for patients with vision impairment, and that interventions were required to address the needs of this population [10]. To increase the likelihood of an intervention being successful, the United Kingdom's Medical Research Council (MRC) advocates utilising the best available evidence and appropriate theory [11, 12]. A theory can be defined as ‘a set of concepts and/or statements with specification of how phenomena relate to each other. Theory provides an organising description of a system that accounts for what is known, and explains and predicts phenomena’ [13]. Apart from McCann et al., who used a limited number of questions derived from the Health Belief Model and the Theory of Planned Behaviour in exploring the behaviour of older patients with vision impairment [1], existing literature is limited by a lack of reliance on theory to help understand the behaviour of patients with vision impairment.

This study was part of a larger project, the overall aim of which was to develop an intervention to support safe and effective medicine use for patients with vision impairment in a hospital and/or community pharmacy setting. Previous work utilised the Theoretical Domains Framework (TDF) to understand the views of pharmacists and factors influencing their behaviour when providing services to patients with vision impairment [14]. The aim of the current study was to identify potential determinants of medicine use from the perspective of patients with vision impairment and their caregivers to help develop a theory‐informed basis for future intervention. The study objectives were to identify the barriers and facilitators to obtaining and taking medicines from the perspectives of patients and their caregivers and identify the key areas that could be targeted by a future intervention.

## 2 Methods

The theoretical foundation for developing effective interventions for patients with vision impairment can be enhanced by utilising the TDF to better understand the factors that influence medicine use from the standpoint of patients and their caregivers. The TDF was developed in an attempt to address the plethora of theories and difficulties associated with selecting the most pertinent for exploring a given behaviour [15]. It comprises 128 theoretical constructs from 33 different psychological theories of behaviour [16]. Constructs were originally grouped into 12 domains (TDF 1) by Michie et al. [15] and later expanded to 14 domains (TDF 2) by Cane et al. [17].

The study employed semi‐structured interviews based on TDF 2 to facilitate in‐depth exploration of the experiences of visually impaired patients and their caregivers in relation to two target behaviours: obtaining and taking medicines. Considering the anticipated challenges associated with organising logistics (e.g. timing and venue) for focus groups, individual interviews were preferred. Interviews were also chosen because they enabled an exploration of this group's diversity in terms of degree of impairment, strengths and needs [18]. This study was reported according to the Consolidated Criteria for Reporting Qualitative Research (COREQ) checklist [19] (Supporting Information S1). An ethics statement detailing ethical approvals and consent process is included at the end of the article.

### 2.1 Consultation With Stakeholders

Before determining the best sampling and recruitment strategy, meetings and/or telephone discussions were conducted with several stakeholders, namely two ophthalmologists, two optometrists specialising in low‐vision services, and an administrator at the ‘National Association of the Blind’ (Kafeef) in Riyadh. These discussions enhanced the research team's understanding of vision impairment, aided decisions on the practicality and logistics for identifying and recruiting patients, assisted with formulating patient eligibility criteria and helped with networking with other key individuals who could provide access to patients. Patients were not consulted before the design of study procedures due to time constraints.

### 2.2 Participants and Recruitment Strategy

Patients over the age of 18 with vision impairment defined according to the World Health Organization (WHO) classification of severity of vision impairment (ICD‐10 version 2016) [20] as having moderate or severe vision impairment, or blindness were eligible to participate in this study. Patients had to be taking a minimum of three medicines on a regular basis for at least the previous month and have no cognitive or hearing defects that would impact their ability to participate in an interview. Patients who used their medicines independently or those who required the assistance of a caregiver were considered eligible.

A purposive sampling technique was adopted to ensure diversity in participants, i.e. males and females of differing age; with different causes, severity and duration of vision impairment; who had different medical conditions; and who took different medications. Patients were recruited through two main approaches:

*Approach 1:* Patients were recruited from four low‐vision clinics (which provide rehabilitation services for people with vision impairment) operating in governmental hospitals in Riyadh, Saudi Arabia. One researcher (B.K.) identified potentially eligible participants by screening patients' medical records. In some instances, the optometrist running the clinic also facilitated recruitment by identifying eligible patients who were then screened by B.K. The researcher (B.K.) then approached eligible patients (and caregivers) and provided them with study information in an alternative format, i.e. larger font or Braille at the participant's request.

*Approach 2:* To ensure sample diversity beyond the groups of patients who attended low‐vision clinics, patients were also recruited through Kafeef, a Riyadh‐based nonprofit organisation that provides social, psychological and educational programs for patients with vision impairment and their families. Kafeef initially supplied the research team with a list of potentially eligible participants. However, all patients in the initial list were not eligible, as they did not fit the medicines use criterion. Subsequently, Kafeef allowed B.K. to screen members' files (anonymised) to identify potentially eligible patients who were then contacted and sent study information as previously described.

Eligible patients and caregivers were followed up by telephone after 1 week of initial contact to formally recruit them to the study. Recruitment and interviewing continued until theoretical saturation was reached, i.e. until no significant new themes emerged from the analysis of the interviews. Saturation was considered separately for patients' and caregivers' interviews.

### 2.3 Conducting the Interviews

Semi‐structured interviews were conducted using two separate interview topic guides, one for patients and one for caregivers (Supporting Information S2). The topic guides were developed based on the 14‐domain TDF 2 [17]. Patients' interviews explored their experiences with obtaining and using their medicines and what might help or prevent them from obtaining and taking medicines as prescribed. Similarly, caregivers' interviews sought to understand their experiences with helping patients obtain and take their medicines and what difficulties they faced in helping patients with their medicines.

Interview guides were written in English, translated into Arabic by a professional translator and the translation was verified by two researchers (B.K. and S.A., native Arabic speakers and fluent in English). Draft interview guides were piloted with two patients and their caregivers which provided an estimation of the interview duration (average of 60 min for a combined patient/caregiver interview). Minor changes in wording were made based on their feedback.

Following receipt of consent, patient/caregiver interviews were carried out face‐to‐face or over the telephone based on the participant's preference. Patients and their caregivers were interviewed at the same time by one researcher (B.K.) who had been trained in qualitative research methods. Each participant received an honorarium of 200 SAR for their time. All interviews were audio‐recorded (using SONY UX560F Digital Voice Recorder), transcribed verbatim manually, with all identifiers removed and replaced with codes, e.g. PT01 (Patient 1), CG01 (Caregiver 1). Transcripts were then translated by a professional translator and B.K. reviewed all translated transcripts for accuracy.

### 2.4 Data Analysis

Separate analysis, using NVivo 12 [21] was undertaken for the patient and caregiver interviews because questions for patients and caregivers focused on different, but closely related behaviours. Initially, the framework method [22] was used to deductively analyse the data, where the 14 TDF domains served as the main coding labels [17]. Content analysis was then conducted, where subcodes were used to identify the barriers and facilitators of medicine use within each TDF domain from the perspectives of patients and caregivers. Initially, three transcripts (10%) were independently coded by both B.K. and another researcher (H.B., or S.A. or C.H.). Coding was then discussed by the research team to compare and refine codes and agree on the final framework. B.K. subsequently carried out the coding of the remainder of the interviews (with ongoing discussions within the team) using NVivo 12 before importing them into a Microsoft Excel spreadsheet to generate a framework matrix. Key TDF domains were identified by assessing the relevance/importance of domains using three criteria: relative frequency of themes, existence of conflicting themes and the perceived strength of impact of the theme on the target behaviour [16]. The feasibility of addressing barriers/facilitators in a future hospital and/or community pharmacy intervention and the overall project timeline were considered. A consensus‐based approach within the research team was utilised to agree on final decisions.

## 3 Results

### 3.1 Participant Characteristics

A total of 213 patients were screened from low‐vision clinics and a further 238 were screened from Kafeef. Thirty‐eight patients were recruited, with eight patients dropping out or being excluded due to one of the following reasons: not responding to multiple requests to set an interview time, dealing with family/other issues, or a caregiver indicating that the patient would not be able to participate. A total of 12 patient/caregiver dyads and 18 individual patients were interviewed for the study. Theoretical saturation was achieved by the 30th patient and the 12th caregiver interviews as no new themes emerged at that point. Three patient/caregiver dyads and two patient interviews were conducted face‐to‐face. All other interviews were conducted over the telephone. Interview duration ranged from approximately 27 min to 100 min. Participants' demographic characteristics are shown in Table 1.

**Table 1:** Participants' characteristics.

Patient code	Patient age (years)	Patient sex	Category of vision impairment^a^	Duration of vision impairment (years)	Patient level of education	Place of residence	Caregiver code	Caregiver relationship to patient
PT01	27	Female	4 Blindness	27	Secondary school	Central region	**CG01**	Mother
PT02	21	Female	3 Blindness	21	Secondary school	Central region	**CG01**	Mother
PT03	82	Female	4 Blindness	1	Limited literacy skills	Central region	**CG02**	Daughter
PT04	88	Female	1 Moderate VI	88 (Left eye), 20 (Right eye)	Limited literacy skills	Central region	**CG03**	Daughter
PT05	72	Female	1 Moderate VI	3	Reads and writes	Southern region	N/A	
PT06	74	Female	1 Moderate VI	4	Limited literacy skills	Central region	**CG04**	Daughter
PT07	57	Female	4 Blindness	54	Bachelor degree	Central region	N/A	
PT08	72	Female	4 Blindness	20	Limited literacy skills	Central region	N/A	
PT09	58	Female	1 Moderate VI	1	Limited literacy skills	Central region	N/A	
PT10	32	Female	2 Severe VI	3	Primary school	Western region	N/A	
PT11	61	Female	1 Moderate VI	15	Limited literacy skills	Central region	**CG05**	Daughter
PT12	35	Female	1 Moderate VI	1	Bachelor degree	Central region	N/A	
PT13	46	Male	2 Severe VI	40	Primary school	Central region	N/A	
PT14	71	Male	1 Moderate VI	15 (Left eye), young age (Right eye)	PhD degree	Central region	N/A	
PT15	66	Female	2 Severe VI	11	Limited literacy skills	Central region	N/A	
PT16	60	Male	1 Moderate VI	4	Secondary school	Central region	N/A	
PT17	37	Male	5 Blindness	2.5	Primary school	Northern region	**CG06**	Wife
PT18	19	Male	2 Severe VI	19	Secondary school	Western region	**CG07**	Mother
PT19	62	Female	2 Severe VI	35	Diploma	Northern region	N/A	
PT20	49	Female	1 Moderate VI	8	Secondary school	Central region	N/A	
PT21	34	Male	1 Moderate VI	20	Diploma	Central region	N/A	
PT22	36	Female	4 Blindness	26	Bachelor degree	Western region	N/A	
PT23	32	Male	2 Severe VI	27	Secondary school	Eastern region	**CG08**	Wife
PT24	20	Female	2 Severe VI	20	Secondary school	Central region	N/A	
PT25	74	Female	1 Moderate VI	> 20	Limited literacy skills	Southern region	**CG09**	Daughter
PT26	66	Female	1 Moderate VI	2	Limited literacy skills	Central region	**CG10**	Son
PT27	34	Female	1 Moderate VI	Young age	Intermediate school	Western region	N/A	
PT28	48	Female	3 Blindness	1.5	Primary school	Central region	**CG11**	Son
PT29	68	Male	1 Moderate VI	2	Intermediate school	Eastern region	N/A	
PT30	52	Male	1 Moderate VI	40	Bachelor degree	Southern region	N/A	

*Abbreviations*: CG = caregiver, N/A = not applicable, PT = patient, VI = vision impairment.

^a^ Based on ICD‐10, 2016, which classifies the severity of vision impairment according to visual acuity into seven categories: category 0 for mild or no vision impairment, category 1 for moderate vision impairment, category 2 for severe vision impairment, and categories 3, 4 and 5 for blindness.

### 3.2 Identification of Barriers and Facilitators That Influenced Patients' and Caregivers' Behaviours

Patients identified several barriers and facilitators that influenced how they obtained and took their medications. Many of the barriers and facilitators identified by caregivers resonated with those reported by patients. ‘Environmental context and resources (ECR)’ and ‘Social influences’ were the two most frequently coded domains. Several barriers and facilitators were reported repeatedly under different TDF domains. Detailed tables of barriers and facilitators identified by patients and caregivers across the 14 TDF domains, together with illustrative quotes, are presented in Supporting Information S3.

Patients believed it was their responsibility to use their medications, but some felt they were not able to be totally independent because of the vision impairment. Caregivers believed it was their role to assist patients with their medications although one caregiver wished that the patient (a young son) would be more responsible for his own medications (Domain: ‘Social professional role and identity’):

…I rely on them [family] to help me, because as a blind person, I mean I cannot be totally independent if there is no Braille writing…

(PT01)

…I'd like him to be attentive, I mean. Attentive on his own, I mean. […] I'd tell him ‘Put a reminder for yourself on the mobile phone if you, like, have a [eye] drop’ […] I mean ‘I will not be there for you forever, your father will not be there for you forever. Depend on yourself!’

(CG07)

Generally, participants demonstrated very good knowledge of the uses, dosing and administration times of medications (Domain: ‘Knowledge’). Patients and caregivers usually referred to medications by indications, e.g. ‘the one for lipids’. Many patients identified their medications by distinguishing features such as shape or colour which led to problems when different brands were dispensed by pharmacists. Many patients and caregivers could not provide the names of their medications. Those who did so, usually younger educated participants, struggled with their pronunciation to the point that some were unidentifiable by the interviewer. One patient identified medication names being in English as a barrier:

Vitamin D comes in pills that are somewhat like beads. I mean I will know them … The folic acid is yellow, I will just recognise it.

(PT01)

[I recognise them] by the box […] I don't know English, you see. […] If it [name] was in Arabic, I'd know it.

(PT16)

Some patients had little knowledge about side effects. This, at times, did not appear to deter patients from using their medications because they felt ‘compelled’ to use them (Domain: ‘Emotion’) and were aware of their benefits (Domain: ‘Beliefs about consequences’). However, fear of perceived side effects sometimes led to patients stopping their medications:

Well, I don't know about them [side effects]. I take them and know nothing.

(PT11)

Because, because people say it causes umm…in, in the ear, like you won't hear well… […] cholesterol [medicine] causes this in the ears so I thought: […] ‘I've lost my sight. Should I lose my umm hearing too! Better get rid of it’.

(PT10)

Failure to identify expiry dates led, in one instance, to the patient taking an expired medication which compromised the patient's clinical outcomes. However, a number of patients reported using mobile telephones, magnifiers (Domains: ‘Skills’ and ‘ECR’), or relying on caregivers or other family members (Domain: ‘Social influences’) to help with checking expiration dates:

The expiration date, I either make my brother, like, read it for me, or get the mobile phone and enlarge with it.

(PT21)

A number of patients and caregivers felt they received limited information about medications and identified the need for further information:

…You'd find written ‘Take one pill for 90 days.’ Ok, a pill for 90 days, I don't know what this is. Why don't you tell me, well, after meal, before meal […] there are no instructions…

(PT16)

…Yeah, of course we want, like, to get more information.

(CG03)

The ‘Skills’ domain was one of the areas that demonstrated the diverse abilities of visually impaired patients. Patients with residual vision relied on shapes/colours to identify their medications, while those who found it difficult to recognise these relied on their sense of touch. With prolonged use of medications, patients have developed unique methods to identify and use medications. This included making their own marks on medication packs, relying on their sense of hearing by listening to clicks to measure the dose on insulin pens and keeping medicines at separate places or in separate bags. Many caregivers relied on teaching patients how to use their medications or organising them in commercially available pill organisers or DIY (‘Do it yourself’) organisers they created out of regular boxes to meet their needs (Domains: ‘Skills’ and ‘ECR’). These organisers also served as a method to monitor medication use (Domain: ‘Behavioural regulation’). Interestingly, some patients and caregivers thought that using pill organisers would lead to errors, reduce the efficacy of medicines or lead to medication contamination:

It is basically obvious by its shape. And even when I…like when the light is off or something. I hold it and feel it. Xolamol [dorzolamide and timolol] is a little short and wide. Iopidine [apraclonidine], no, it is short and narrow.

(PT24)

They [family] come and make it [pill organiser] for me and prepare everything for me in this bag and put it aside in the drawer. This drawer is beside my bed.

(PT28)

I mean, we taught her, this green box is aspirin. Yeah, and the [one for] diabetes we taught her that this big one she has to break it, one with breakfast and one with dinner.

(CG03)

ECR was the most frequently coded domain in both sets of interviews with barriers/facilitators identified under this domain influencing a number of other domains. For example, patients' beliefs about their capability in obtaining and taking their medications (Domain: ‘Beliefs about capabilities’) were mostly conditional on factors such as the availability of medicines, vision aids and Braille information (Domain: ‘ECR’). Patients living outside major cities, repeatedly mentioned unavailability of medicines as a barrier under different domains. Medication delivery services were useful to such patients and suspension of these services was a barrier to obtaining their medications:

Some medications do not come here to us in villages […] So, it is difficult for you to find it. You need to go to the big city…

(PT12)

A number of barriers under the ‘ECR’ domain were specific to medicines' labels or the pharmacy environment. Several patients reported that the queuing system at the pharmacy, whereby patients received a number from an electronic device and had to watch a screen for that number before speaking to the pharmacist, created problems. Some could not use the electronic device because they could not see which buttons to push and almost all had difficulty seeing numbers as they appeared on the screen (Domain: ‘ECR’):

…Sometimes the number would come up, I cannot see the screen. I cannot see it to read or anything.

(PT20)

The lengthy process, the need for repeated visits, and the limited quantity of dispensed medications were also identified as barriers by a number of participants. One caregiver described how the unavailability of medications at the pharmacy forced him to ask for multiple exemptions from work to obtain medications for the patient as they became available. However, some caregivers found the process of ordering medications through smartphone applications or telephone calls useful and straightforward (Domain: ‘ECR’). Only three patients in the current study knew Braille and only two of those actively utilised it in relation to medication use (Domains: ‘ECR’ and ‘Knowledge’). Younger participants made use of accessibility features in their smartphones including the camera, voice commands and alarms (Domains: ‘Skills’ and ‘ECR’):

I have a speaker, screen reader […] in the iPhone.

(PT01)

…Before the App [sic], I used to go to the pharmacy and give them a paper where I write all the needed medications […] and leave the paper there and go. After 24 hours, I'd go back again. They either approve it or don't approve it.

(CG11)

Participants expressed positive intentions and emotions regarding medication use (Domains: ‘Intentions’ and ‘Emotions’) and generally believed in the benefits of medications (Domain: Beliefs about consequences), which encouraged them to use medicines as prescribed (Domains: ‘Reinforcement’ and ‘Goals’). Some participants, however, felt discouraged by negative emotions (e.g. feeling bored) and the side effects or number of medicines. One patient described being embarrassed by having to use medicines in public (Domain: ‘Emotion’). The priority of medications varied depending on what participants considered more important (Domain: ‘Goals’):

I mean, I have moments of feeling bored of using [medications]. But, I'd go back and see that the benefit of using is more important than the boredom I'm feeling. So, it gives me a motivation to continue.

(PT30)

Less important because I feel they [eye drops] are not useful […] Every time I ask him [husband] ‘Do you feel there is improvement?’, he'd say ‘No, it is the same.’ And I feel he is the same. He is the same. There is no change!

(CG06)

Having and maintaining a routine was regarded by participants as a major facilitator (Domain: ‘Memory, attention and decision processes [MADP]’). Other facilitators included using alarms, organising medicines in a specific manner, and paying attention to medicines deemed to be of greater importance. One caregiver (mother) who cared for three patients with vision impairment (daughters), two of whom were also interviewed, indicated negative effects on her own health, being diagnosed with depression and diabetes mellitus. She also reported the impact on both hers and the family's social lives and the feeling of tiredness which made her more careful about medication administration to avoid her daughters' hospitalisations (Domains: ‘Emotions’, ‘MADP’ and ‘Reinforcement’):

I mean, I don't set a mobile phone [alarm] or set anything, no, I don't…I already got used to them.

(CG07)

The tiredness…the tiredness, the tiredness. By God, I am honestly tired! I've got diabetes, took psychiatric medication. By God, I am tired!

(CG01)

‘Social influences’ was frequently coded throughout the interviews. All visually impaired patients, even those who identified themselves as independent during the screening process, required some sort of support by family members. Caregivers assisted patients with ordering and collecting medications, checking expiry dates, organising medications, administering medications (e.g. eye ointments and syrups) and reminding patients to take their medications. Caregivers relied on other family members or housekeepers to assist with a number of aspects particularly when they were not available to assist patients (Domain: ‘Social influences’). Caregivers did not discuss lack of confidence in their own abilities but focused on the patient's ability to manage aspects of medication use (Domain: ‘Beliefs about capabilities’). Patients' reluctance to take medications was identified by caregivers as a barrier under multiple domains including ‘Beliefs about capabilities’, ‘Optimism’, ‘Reinforcement’, ‘Goals’ and ‘Social influences’:

…He [patient] is not accepting [eye drops]. He is irritated I mean, very irritated by them. With a fight, I swear it is with a fight I mean …for him to put the thing.

(CG07)

My mother is illiterate. […] That is what she can do, like ‘Put it in the box and I'll take it’.

(CG04)

…Maybe four medicines, all of them were syrups. These, I had to be careful that I give it to them myself. […] Because, you know, it's hard to draw the milli [millilitres] and so on. They can't see.

(CG01)

Patients assumed that healthcare professionals (including pharmacists) knew about their impairment from their medical files, the type of medications they received or their appearance. Moreover, patients did not disclose their impairment to healthcare professionals as they assumed those not specialising in ophthalmology would not be interested in knowing such information. None of the patients in this study used the white cane commonly used by visually impaired people:

It [knowing about VI] is not his business, he [pharmacist] just dispenses the medication and that is it.

(PT20)

He [pharmacist] knows [about VI] of course. Because of the medications I take from him and so on.

(PT29)

Physicians were held in high regard by patients and were perceived to be knowledgeable about medicines. Conversely, patients thought the pharmacist's role was to just dispense medications and reiterate the physician's instructions. Although a number of participants reported favourable interactions with pharmacists, several were unsatisfied with services provided. A few patients perceived that hospital pharmacists were busy and had many patients to serve and consequently did not feel comfortable discussing issues with them out of respect to other patients:

I need the pharmacist only for dispensing medication. But, for the instruction process or the essentials, the treating doctor.

(PT30)

Well, I don't remember that a pharmacist sat and told us anything. […] I mean I'd get the medication, and he'd tell me that the instructions are on the paper.

(CG11)

Yeah, but why I don't ask the pharmacist at the hospital? Because […] there would be many people waiting and there are people who cannot… older people, cannot bear it, you know?

(PT22)

Facilitators identified under the ‘ECR’, ‘MADP’ and ‘Social influences’ domains were linked to the ‘Behavioural regulation’ domain. Participants identified pill organisers, having a routine, family support, and using mobile phone alarms as methods that helped monitor the patients' medication use:

…If I'm doubtful…doubtful about not taking something […] They [family] would be around, [I'd ask] “Did I take this medication or not?”…They'd tell me.

(PT10)

…So, I take breakfast and go take the medicines and that's it. And when I have dinner at night, around sunset, I take the rest.

(PT05)

I'd call her at half past six and say, ‘Go take the medicine’ and she takes her medication. The housekeeper would be with her. […] I'd call the housekeeper and ask her, she'd say ‘Yeah, she took the medication’.

(CG01)

Patients linked their optimism about overcoming problems with medication use with their religious beliefs that ‘God will help’ and were less optimistic when they had previous negative experiences with medications. Caregivers linked their optimism with patients' abilities and their state of vision (Domain: Optimism):

Well, look, I tried… […] I had to put [the ointment] on the tip of a finger and put it at the bottom of the eye, but…it does not work! It does not work! That's why I stopped it the three or 4 days…

(PT30)

Yeah, if his sight returns or if for example there was education […] someone teaching him how to read, how to tell things apart…

(CG06)

### 3.3 Identification of Key TDF Domains

The 14 key TDF domains were all considered relevant to the target behaviours. They were repeatedly coded across interviews and were found to have a strong influence on patients' and caregivers' behaviours. Of these, the following domains were considered ‘key domains’ that could be targeted by a future intervention: ‘Knowledge’, ‘Skills’, ‘MADP’, ‘ECR’, ‘Social influences’ and ‘Behavioural regulation’. The ranking of domains as ‘key’ or ‘less important’ is outlined in Table 2.

**Table 2:** Importance of TDF domains identified from patients'/caregivers' interviews.

Category	Domains	Comments
Key domains	Knowledge Skills MADP ECR Social influences Behavioural regulation	ECR and social influences were the most coded domains in both sets of interviews. Knowledge and skills domains were also commonly coded in both sets of interviews. Barriers identified under MADP and behavioural regulation domains were found to be common and recurrent across interviews.
Domains of less importance	SPRI Beliefs about capabilities Optimism Beliefs about consequences Reinforcement Intentions Goals Emotion	SPRI: Patients/caregivers agreed that aspects relating to obtaining and taking medicines were their responsibility. Beliefs about capabilities: Almost all barriers/facilitators under this domain were linked to ECR. Optimism: Some barriers and facilitators under this domain were linked to ECR and social influences. Beliefs about consequences: Patients/caregivers were well aware of the benefits associated with medication use. Some of the barriers identified under this domain were linked to the knowledge domain. Reinforcement: Barriers/facilitators were linked to other domains, including goals, emotions and social influences. Intentions: Patients/caregivers had positive intentions. Identified barriers were all linked to ECR. Goals: Patients/caregivers considered medications important. Identified barriers were linked to knowledge and MADP. Emotion: Some barriers and facilitators were linked to knowledge and ECR.

Abbreviations: ECR = environmental context and resources, MADP = memory, attention and decision processes, SPRI = social/professional role and identity.

## 4 Discussion

This study utilised a systematic process to identify the determinants of visually impaired patients' behaviours of obtaining and taking medicines and the behaviour of caregivers in assisting patients with these processes. The TDF was used as the theoretical lens through which these behaviours were examined. This study also involved identifying key TDF domains for the target behaviours. These can potentially be used to develop an intervention to support safe and effective medicine use for this patient population.

Despite their vision impairment, patients seemed to cope well with many difficulties they encountered while obtaining and taking medications. It was also apparent that caregivers played a major role in assisting visually impaired patients with medicines. Patients with vision impairment and their caregivers identified a wide range of barriers and facilitators under each TDF domain. While many of these were common among participants, it was evident that patients with vision impairment cannot be treated as a homogeneous group. This could be partly attributed to the varying levels of vision impairment but also due to individual patients' unique abilities, beliefs, needs and experiences. This has been previously reported in the literature where visually impaired individuals objected to being ‘clumped together in large groups of visually impaired people with whom all they shared was owning the same diagnostic label’ [18].

Consistent with the findings of Zhi‐Han et al., who reported that 58% of visually impaired respondents did not know the names of their medications [3], many patients and caregivers in the current study could not provide these details and medications were referred to in terms of indications. While this was not identified as a barrier according to patients or caregivers, it could potentially lead to medication errors. Issues where changing the brands of medicines created problems for patients who relied on shape or colour to identify them have been also previously reported by Smith et al. with patients with vision impairment in Scotland [8]. Identification of expiration dates appears to be one of the major problems facing patients. A survey of blind patients in Saudi Arabia showed that 92% of participants had issues identifying expiration dates and 90% required the assistance of a sighted person [2].

Pill organisers were noted as useful aids by both patients and caregivers. The use of such organisers particularly by older patients was found to ease medication management, enhance transportability of medications and improve adherence and clinical outcomes [23]. In line with caregivers' opinions, the use of pill organisers has been associated with several limitations such as their effect on medications' stability when removed from original packaging, and the risk of dispensing errors during the filling process [24, 25, 26]. Issues with pharmacy queuing systems identified in this study have also been reported by Phongpunpisand et al. [27] in relation to hospital pharmacy services for visually impaired persons in Thailand. It has been suggested that an audio calling system eases communication with visually impaired patients at the pharmacy as they may not be able to see a visual display [28]. While providing information in Braille was recommended by some guidelines for visually impaired patients [28, 29, 30], only three patients in the current study knew Braille and only two actively utilised it in relation to medication. This contrasts with results of a recent survey of visually impaired people in Saudi Arabia, which found that 89% of the participants knew Braille and 86% thought that Braille labelling was the ideal method to dispense medications to the visually impaired [31]. These contradictory findings may have a number of explanations. The online survey was distributed through associations for blind people and blind social media influencers [31]. Being a member of one such association could grant people more access to support services such as Braille learning workshops that non‐members may not be aware of or have access to. Moreover, approximately 70% of the survey respondents had a university degree which may mean they were more likely to have learned Braille. The use of multiple smartphone features reported by younger patients in this study reflects earlier studies reporting that people with vision impairment have been increasingly using these devices for daily tasks such as navigation, emailing, social networking and even for academic education [32, 33]. The accessibility features and apps embedded within smartphones that are specifically designed to support visually impaired people [32] make them an attractive option to include in a future intervention to assist patients with medication use. Easley et al. indicated that visually impaired patients were positive towards using mobile phones for medication management and were willing to experiment with novel technologies as long as these met their requirements [5].

The findings that patients and caregivers presumed that healthcare professionals (including pharmacists) were aware of their impairment, that patients did not disclose their impairment to healthcare professionals, and that none of the patients reported the use of a white cane are concerning. These may contribute to pharmacists failing to identify those patients when they present to pharmacies, which has been previously reported [34], and subsequently failing to provide appropriate services tailored to their needs. Participants' perspectives regarding the superiority of physicians' knowledge and the limited role of pharmacists were consistent with previous data. Other studies have shown that physicians were viewed as superior to pharmacists in knowledge and training [35, 36]. Patients have also described a pharmacy as a dispensary and lacked awareness of extended pharmacy services [35, 37]. In Middle Eastern countries, only 30% to 50% of patients had trust in pharmacists' advice about medications [36]. In Saudi Arabia, one reason identified for lack of trust in community pharmacists was a perception that they were business‐oriented rather than patient‐oriented [38]. Prior research with visually impaired people indicated that most identified physicians as the preferred source of medication information [39, 40] and also believed physicians were a more reliable source of information than pharmacists [2]. Koo et al. reported that visually impaired individuals had difficulties consulting pharmacists when other people waited to be served [6]. This aligns with the current study findings where participants believed pharmacists were busy, which prevented them from requesting additional information about medicines.

The role of caregivers highlighted in this study reflects earlier findings [1, 2, 5, 40, 41], yet a deeper understanding is provided of factors influencing their own behaviours when facilitating medication use for patients with vision impairment. A recent systematic review and meta‐analysis reported that caregiving to patients with vision impairment was associated with significant burden, which negatively affected the health, social, psychological and financial situations of caregivers [42]. In the current study, some caregivers described negative effects on their health, their social life or their work. These findings are consistent with those of a study on the burden perceived by caregivers in Saudi Arabia, where nearly three‐quarters of those investigated had work‑related issues and depression or mood swings [43]. The need for further information about medications expressed by a number of caregivers is in accordance with a recent study where caregivers of people with vision impairment advocated for improvements in information provision and other forms of support (e.g. practical, emotional and social) [44].

There is a need to raise pharmacists' awareness of the many challenges that patients encounter and of strategies to support patients and their caregivers. Pharmacists should be aware that patients with vision impairment may not be easily identifiable and recognise that a ‘one size fits all’ approach to medication dispensing and counselling will fail to address some of the unique needs of these patients. It was clear from the current study findings that any future interventions for patients with vision impairment should be carefully tailored and individualised. The variation in patients' capabilities and extent of vision impairment translated into differing needs, types of support and methods for information delivery. Providing information regarding medication dosages, instructions, expiry dates and major side effects in Braille was shown to significantly reduce medication errors and challenges with medication administration [45], although some participants may prefer tactile methods over Braille [46]. Larger print medication labels were also found to be preferred by visually impaired people and were likely to decrease the barriers they encountered when reading labels [47, 48]. However, the findings from the current study suggest that providing information in Braille, enlarging the font size, or utilising tactile methods to convey medication instructions are not always universal solutions. These findings support the recommendation made by a number of guidelines regarding the importance of asking patients about their needs and how they prefer the information to be delivered to them [28, 30, 49]. The key TDF domains identified in this study provide a good start for developing future interventions.

### 4.1 Strengths and Limitations

The study reported here followed the recommendations of the UK's MRC guidance [11, 12] to develop a theory‐informed in‐depth understanding of patients' and caregivers' behaviour. It is one of the few studies that has utilised a qualitative methodology to help understand the medication use behaviour of patients with vision impairment. To the authors' knowledge, it is the first study to present a thorough examination of caregivers' experiences. The sample was relatively large and included younger and older patients with varying degrees of vision impairment. While all patients in this study were recruited from Riyadh city (Central Region), several were residing in other regions in Saudi Arabia, providing unique insights into the experiences of patients living in areas where available resources may differ from the capital. Double coding of 10% of the interviews, regular debriefings, providing ‘thick descriptions’ and maintaining an audit trail of data analysis throughout the study enhanced the trustworthiness and authenticity of the research findings. Participants were aware that B.K. was a lecturer and a PhD student. The affiliation with an academic institution and having a pharmacy background helped to enhance B.K.'s credibility with the participants but may have also resulted in more favourable responses (social desirability bias). The research team was aware of the ethical responsibility involved in conducting research with a vulnerable population and took precautions to ensure the accessibility of study information packs and adherence to IRB procedures for obtaining consent. Reflexivity was employed through notes and memos being recorded both after each interview to capture first impressions and thoughts and during the analysis process to document ideas and challenges. Before piloting, interview topic guides were carefully constructed and revised to avoid leading questions. To ensure the quality of reporting, the study has been reported in accordance with the COREQ checklist [19]. Member checking was not carried out because of time constraints. Although patients/caregivers were asked about causes of vision impairment and number and types of all medications used, they did not always report this completely or accurately. Medical records were limited to eye diagnoses and associated medications and rarely contained information on other diseases or medications.

## 5 Conclusion

This study outlined the use of the TDF to explore the barriers and facilitators that influenced the medicine use behaviours of patients with vision impairment and their caregivers. The two most coded domains were ‘ECR’ and ‘Social influences’ with a number of important barriers and facilitators also identified under other domains, e.g. ‘Knowledge’, ‘Skills’, ‘MADP’ and ‘Behavioural regulation’. Identification of factors that influence the behaviour of visually impaired patients and their caregivers is a unique contribution to the literature, which has typically focused on identifying challenges encountered by this patient population. Future research should focus on developing an intervention that aims to improve the processes of obtaining and taking medications for patients with vision impairment and their caregivers.

## Author Contributions

**Basma Y. Kentab:** conceptualisation, methodology, data curation, investigation, formal analysis, project administration, writing – original draft, validation. **Heather E. Barry:** conceptualisation, methodology, formal analysis, supervision, writing – review and editing, project administration, validation. **Sinaa A. Al‐Aqeel:** conceptualisation, methodology, formal analysis, supervision, writing – review and editing, project administration, validation. **Carmel M. Hughes:** conceptualisation, methodology, formal analysis, supervision, project administration, writing – review and editing, resources, validation.

## Ethics Statement

This study was performed in line with the principles of the Declaration of Helsinki. Approval was granted by the Institutional Review Boards of King Saud University (Reference No. 19/0090/IRB), King Khaled Eye Specialist Hospital (Reference No. RD/26000/7076‐19) and King Fahad Medical City (Reference No. H‐01‐R‐012).

## Consent

Local Institutional Review Board's (IRB) procedures were followed to obtain consent from participants with vision impairment and those with limited literacy skills. Written or electronic informed consent were obtained from all participants (with support from family members where appropriate) before commencing data collection.

## Conflicts of Interest

The authors declare no conflicts of interest.

## Supporting information

Supporting information.

Supporting information.

Supporting information.

## Data Availability

The data that support the findings of this study are available on request from the corresponding author (C.H.). The data are not publicly available as the contents could compromise research participants' confidentiality.
