# Supplementary material for: Barriers and Facilitators to Medicines Use in Patients With Vision Impairment: A Theory‐Informed Qualitative Study of Patients and Caregivers
Source: Health Expect. 2025 Mar 21;28(2):e70234. doi: 10.1111/hex.70234 (PMC11926560; doi:10.1111/hex.70234)
Supplement: Supplementary file 1 — Supporting information. [file HEX-28-e70234-s002.pdf]

Barriers and facilitators to medicines use in patients with vision impairment: A theory-informed qualitative study of patients and caregivers

The Consolidated criteria for reporting qualitative research (COREQ) checklist

No. Item	Guide questions/description	Comments
**Domain 1: Research team and reflexivity**		
Personal Characteristics		
**1. Interviewer/facilitator**	Which author/s conducted the interview or focus group?	One researcher (BK) conducted all interviews.
**2. Credentials**	What were the researcher's credentials? e.g. PhD, MD	BK had a BSc. in Pharmaceutical Sciences and MSc. in Clinical Pharmacy and was a PhD student at the time of the study. HB, SA, and CH have a PhD in Pharmacy.
**3. Occupation**	What was their occupation at the time of the study?	BK was a lecturer at King Saud University and a PhD student at Queen's University Belfast. HB was a Senior Lecturer at Queen's University Belfast. SA was a Professor at King Saud University. CH was a Professor at Queen's University Belfast.
**4. Gender**	Was the researcher male or female?	All research team members were females.
**5. Experience and training**	What experience or training did the researcher have?	BK had undertaken training in qualitative research methodologies. HB, SA, and CH had experience with conducting TDF-based and/or qualitative research.
Relationship with participants		
**6. Relationship established**	Was a relationship established prior to study commencement?	No prior relationship was established prior to the contact about the study.
**7. Participant knowledge of the interviewer**	What did the participants know about the researcher? e.g. personal goals, reasons for doing the research	Participants were aware that BK was a lecturer and a PhD student and that HB, SA, and CH were academic staff supervising BK . Participants were briefed on the purpose of the study.
**8. Interviewer characteristics**	What characteristics were reported about the interviewer/facilitator? e.g. Bias, assumptions, reasons and interests in the research topic	All authors had an interest in the research topic.
**Domain 2: Study design**		
Theoretical framework		
**9. Methodological orientation and Theory**	What methodological orientation was stated to underpin the study? e.g. grounded theory, discourse analysis, ethnography, phenomenology, content analysis	The Theoretical Domains Framework (TDF) underpinned the development of the study. Framework analysis was carried out followed by content analysis.
Participant selection		
**10. Sampling**	How were participants selected? e.g. purposive, convenience, consecutive, snowball	Purposive sampling was utilised.
**11. Method of approach**	How were participants approached? e.g. face-to-face, telephone, mail, email	Participants from low vision clinics were contacted face-to-face and those from Kafeef were contacted by telephone. This was followed by a formal invitation letter.
**12. Sample size**	How many participants were in the study?	12 patient/caregiver dyads and 18 individual patients.
**13. Non-participation**	How many people refused to participate or dropped out? Reasons?	Eight patients dropped out or were excluded due to: not responding to multiple requests to set an interview time, dealing with family/other issues, or a caregiver indicating that the patient would not be able to participate.

No. Item	Guide questions/description	Comments
Setting		
**14. Setting of data collection**	Where was the data collected? e.g. home, clinic, workplace	Patients were interviewed at Kafeef offices, a hospital clinic or over the telephone.
**15. Presence of non-participants**	Was anyone else present besides the participants and researchers?	No.
**16. Description of sample**	What are the important characteristics of the sample? e.g. demographic data, date	Participants were visually impaired and some were assisted by caregivers. Nine patients were males and 21 were females.
Data collection		
**17. Interview guide**	Were questions, prompts, guides provided by the authors? Was it pilot tested?	A topic guide with prompts was developed and used during the semi-structured interviews. The topic guide was piloted with two patients and their caregivers.
**18. Repeat interviews**	Were repeat inter views carried out? If yes, how many?	No repeat interviews were required.
**19. Audio/visual recording**	Did the research use audio or visual recording to collect the data?	Interviews were audio-recorded.
**20. Field notes**	Were field notes made during and/or after the interview or focus group?	BK recorded field notes during and after the interviews.
**21. Duration**	What was the duration of the inter views or focus group?	Interview duration ranged from 27 minutes to 100 minutes.
**22. Data saturation**	Was data saturation discussed?	Data saturation was achieved by the 30th patient interview and the 12th caregiver interview.
**23. Transcripts returned**	Were transcripts returned to participants for comment and/or correction?	Transcripts were not returned to participants.
**Domain 3: Analysis and findings**		
Data analysis		
**24. Number of data coders**	How many data coders coded the data?	Two researchers (BK and HB or SA or CH) coded three interviews initially and then BK coded the remainder of the interviews.
**25. Description of the coding tree**	Did authors provide a description of the coding tree?	Data was reported under the TDF domains and barriers/ facilitators were identified under each domain.
**26. Derivation of themes**	Were themes identified in advance or derived from the data?	TDF domains represented the main themes under which barrier/facilitators were identified from the data.
**27. Software**	What software, if applicable, was used to manage the data?	NVivo® QSR 12
**28. Participant checking**	Did participants provide feedback on the findings?	Future publications will be made accessible to participants.

Barriers and facilitators to medicines use in patients with vision impairment: A theory-informed qualitative study of patients and caregivers

No. Item	Guide questions/description	Comments
Reporting		
**29. Quotations presented**	Were participant quotations presented to illustrate the themes/findings? Was each quotation identified? e.g. participant number	Quotations were presented within the results section with participants given an anonymous code (e.g. PT28, CG10).
**30. Data and findings consistent**	Was there consistency between the data presented and the findings?	See Results and Discussion section. Efforts were taken to present data in a clear and consistent manner highlighting where differences s were identified.
**31. Clarity of major themes**	Were major themes clearly presented in the findings?	
**32. Clarity of minor themes**	Is there a description of diverse cases or discussion of minor themes?	
