# Supplementary material for: Barriers and Facilitators to Medicines Use in Patients With Vision Impairment: A Theory‐Informed Qualitative Study of Patients and Caregivers
Source: Health Expect. 2025 Mar 21;28(2):e70234. doi: 10.1111/hex.70234 (PMC11926560; doi:10.1111/hex.70234)
Supplement: Supplementary file 2 — Supporting information. [file HEX-28-e70234-s003.pdf]

Patients' interview topic guide

Introduction

My name is [Basma Kentab]. I am a lecturer at [King Saud University] and I am conducting this study as part of my PhD studies at [Queen's University Belfast]. Thank you for taking the time to speak with me today.

In this study we are speaking to patients who have problems with their sight and their caregivers to explore how patients get and use their medicines. For the purposes of this study, we describe the caregiver as someone who has contact with the patient with sight problems at least three times a week and provides help to the patient with their medicines. We will also speak to pharmacists to find out about their experiences of providing medicines and talking about medicines to patients who have problems with their sight or their caregivers. We plan to use the information gathered during this study to come up with ways to help these patients get the most out of their medicines.

Have you had a chance to read through the information sheet or have somebody read it to you? Are there any questions that you would like to ask me?

Explaining what will happen in the interview and afterwards

This is your chance to share your experiences in getting and taking medicines and what might help you or prevent you from getting and taking them as prescribed. The interview should last about 1 hour.

I will be recording the interview on a digital recorder, to make sure that we have an accurate record of what is discussed. The recording will be saved on a password-protected computer and only those directly involved in the research study will listen to it. The recording will be typed up word-for-word and any names, locations, or anything else that could identify you or anyone you talk about will be removed. Your participation is voluntary, and you are free to stop the interview at any time or skip any question you don't wish to answer. After we complete the interviews of all participants, we will analyse the information and results may be published in scientific journals and conferences.

I have here your written consent indicating that you agree to participate in the study and have this interview audio-recorded. [check that the written consent form is appropriately signed and dated]

Do you have any questions or would like any additional details before we start? [Answer questions.]

[Turn the digital recorder on]

Demographic information

- How old are you?
- What is your highest level of education?
- Where do you live (city/town)?
- Who do you live with?
- Gender, recruitment venue (low vision clinic and its location/charity) (To be recorded on separate note for each participant)

Background information on visual impairment

- When were you diagnosed with sight problems?
- What was the cause of your sight problems?
- How would you personally describe your sight problems?
  - *Prompts:* Blindness, partial blindness, low vision
- Visual acuity measurement (To be obtained from medical, charity records and recorded on separate not for each patient)

Behavioral elicitation

“Before we begin, it would be useful if you could think about all the medicines that you are taking. Over the next few questions, I will be asking you about your experiences of taking your medicines and any problems you may be having. I will also ask you about any medicines-related services being provided to you by your doctor or pharmacist”

Knowledge

- Approximately, how many medicines are you taking?
- What medicines are you taking?
- What do you know about your medicines?
    - *Prompts*
        - Do you know what every medicine is for?
        - Do you know how to take every medicine? How many times a day and for how long do you need to take them?
        - Do you know about the possible side effects of your medicines?

Skills

- How do you get your medicines?
    - *Prompts*
        - Who prescribed these medicines (a physician from a private hospital, governmental hospital, primary healthcare centre)?
        - Where do you get your medicines dispensed (a hospital, primary healthcare centre, or community pharmacy)?
        - Do you get your medicines by yourself or does somebody help?
        - Do you ever use a community pharmacy to get your medicines (ask if community pharmacy not mentioned above)?
            - How often do you use a community pharmacy to get your medicines? Do you use the same one?
- How do your sight problems affect how you take your medicines? (for example: reading medicines' labels? telling tablets apart? measuring liquid medicines? identifying expiry dates?) (Ask about different dosage forms: tablets, eyedrops, injections, creams)
        - *Prompt:* Does anyone help you take your medicines? What do they help you with?
- Has anyone ever checked to see that you are able to get or take your medicines? Have they offered any advice? What advice did they offer?

- What would you need to help you get your medicines, and to take them in the right way?
    - *<u>Prompts:</u>* Talking insulin pens, different colour packages, braille on package, dispensing medicines in different bags, dispensing in original package not bags or vials

<u>Social/professional role and identity</u>

- What do you think your responsibility is/responsibilities are in relation to your medicines?
    - *Prompts*
        - Is there anything apart from these things that you should be responsible for?
        - Do other people have some responsibilities for your medicines? What are these responsibilities?

<u>Beliefs about capabilities</u>

- In what situations do you feel confident about getting and taking your medicines?
- In what situations do you not feel confident about getting and taking your medicines?
    - *Prompt:* What would help you to overcome these problems or difficulties?

<u>Optimism</u>

- How optimistic are you that you can overcome any problems with your medicines?

- What would make you feel less optimistic about overcoming any problems with your medicines?

<u>Beliefs about consequences</u>

- What do you think are the benefits of taking your medicines as prescribed?

    - *Prompts:*
        - For yourself; caregiver; relatives
        - In general, what are the benefits of patients taking their medicines as prescribed?
        - Can you think of any short- and long-term consequences of taking your medicines as prescribed?

- Can you think of any disadvantages to taking your medicines as prescribed?
- Are the benefits of taking all of your medicines worth the possible disadvantages?

Reinforcement

- What would encourage you to take your medicines?
    - *Prompts*
        - Feeling better, family support
- What would discourage you from taking your medicines?
    - *Prompts*
        - Too many medicines, side effects

Intentions

- Do you intend to get your medicines from your pharmacist and take them as prescribed?
- What would prevent you from getting your medicines from your pharmacist and taking them as prescribed?
    - *Prompt:* Anything related to your sight problems?

Goals

- To what extent is taking your medicines a priority for **you**?
- When do you think it is less important to take your medicines?

Memory, attention and decision processes

- In this question I am interested to know how you remember to take your medicines. Do you have any reminders, prompts or routines?
    - *Prompt:* What is your usual routine? Is there something that/someone who helps you to remember to take your medicines?
- Are there any circumstances in which you might just forget or find it difficult to take your medicines?

Environmental context and resources

- What things would help you to take your medicines?
    - *Prompts*
        - Pillbox, reminders
- What things might prevent you from taking your medicines?

    - *Prompts*
        - Home environment, being away from home

Social influences

- Who would influence your decisions to take your medicines?
    - *Prompts*
        - Family, friends, pharmacist, doctor, nurse, optometrist, other healthcare professional
- Can you tell me more about how they influence/help you?
    - *Prompts:*
        - Is there anything that your pharmacist/pharmacy does that makes it easier for you to take your medicines?
        - Is there anything that your pharmacist/pharmacy does that makes it harder for you to take your medicines?
        - What role, if any, does any other healthcare professional (e.g. doctor, nurse, optometrist) play in helping you take your medicines as prescribed?
- Are all healthcare professionals providing care for you aware of your sight problems?
    - *Prompts*
        - Doctors, nurses, pharmacists
        - If yes – how did they know? If no, why is that?
        - Do you tell people about your sight problem and ask for help?
        - Do you use a white cane?

Emotion

- How does taking your medicines make you feel?
    - *Prompts*
        - Does taking medicines make you feel happy/sad/anxious/stressed?
- How does that influence your decision to take your medicines?

<u>Behavioural regulation</u>

- Do you have something in place that could help you check whether or not you have taken your medicines?
    - *Prompts*
        - For example, a calendar, pillbox
- Can you tell me more about how this works for you?

Future Planning

"The research team is interested in developing a plan to support and try to help patients with sight problems and their caregivers with medicines. It has been useful to hear about your experiences in more detail as this will help us to try and understand what patients and their caregivers are struggling with when it comes to medicines, and therefore what aspects we should target as part of our approach to improve the use of medicines in the future."

- If you were to think about the way in which you get and take your regular medicines, can you tell me:
    - What works well with that process/system?
    - What could be done differently that may make things better?
- If your pharmacist arranged to sit down with you and go through all the medicines that you take, how would you feel about this?
    - What would you like to see happen as a result?
- Is there anything else that a pharmacist could do to help you with your medicines?

Closing the interview

"That brings us to the end of the interview.

Is there anything else about your medicines that we should have talked about, but didn't?

Do you have any additional comments you would like to make?

Thank you very much for making the time to speak with me today."

[Turn the digital recorder off]

Caregivers' Interview Topic Guide

Introduction

My name is [Basma Kentab]. I am a lecturer at [King Saud University] and I am conducting this study as part of my PhD studies at [Queen's University Belfast]. Thank you for taking the time to speak with me today.

In this study we are speaking to patients who have problems with their sight and their caregivers to explore how patients get and use their medicines. For the purposes of this study, we describe the caregiver as someone who has contact with the patient with sight problems at least three times a week and provides help to the patient with their medicines. We will also speak to pharmacists to find out about their experiences of providing medicines and talking about medicines to patients who have problems with their sight or their caregivers. We plan to use the information gathered during this study to come up with ways to help these patients get the most out of their medicines.

Have you had a chance to read through the information sheet? Are there any questions that you would like to ask me?

Explaining what will happen in the interview and afterwards

This is your chance to share your experiences in helping [patient name] get and use their medicines and what difficulties you face in helping them with their medicines. The interview should last about 1 hour.

I will be recording the interview on a digital recorder, to make sure that we have an accurate record of what is discussed. The recording will be saved on a password-protected computer and only those directly involved in the research study will listen to it. The recording will be typed up word-for-word and any names, locations, or anything else that could identify you or anyone you talk about will be removed. Your participation is voluntary, and you are free to stop the interview at any time or skip any question you don't wish to answer. After we complete the interviews of all participants, we will analyse the information and results may be published in scientific journals and conferences.

I have here your written consent indicating that you agree to participate in the study and have this interview audio-recorded. [check that the written consent form is appropriately signed and dated]

Do you have any questions or would like any additional details before we start? [Answer questions.]

[Turn the digital recorder on]

Demographic information

- How old is [patient name]?
- What is [patient name] highest level of education?
- Where does [patient name] live (city/town)?
- Who does [patient name] live with?

Background information on visual impairment

- When was [patient name] diagnosed with sight problems?
- What was the cause of [patient name]'s sight problems?
- How would you personally describe [patient name]'s sight problems?
    - *Prompts:* Blindness, partial blindness, low vision
- How are you related to [patient name]?
    - *Prompts:* Daughter, son, mother, father, brother, sister
- How long the have you been involved with helping [patient name] with their medicines?

Behavioral elicitation

"Before we begin, it would be useful if you could think about all the medicines that [patient name] are taking. Over the next few questions, I will be asking you about your experiences in helping [patient name] take their medicines and any problems they may be having as well as any medicines-related services being provided by the doctor or pharmacist"

Knowledge

- Approximately, how many medicines does [patient name] take?
- What medicines does [patient name] take?
- What do you know about [patient name]'s medicines?
    - *Prompts*
        - Do you know what every medicine is for?
        - Do you know how every medicine should be taken? How many times a day and for how long should they be taken?
        - Do you know about the possible side effects of each medicine?

Skills

- How do you get [patient name]'s medicines?
    - *Prompts*
        - Who prescribed these medicines (a physician from a private hospital, governmental hospital, primary healthcare centre)?
        - Where do you get [patient name]'s medicines dispensed (a hospital, primary healthcare centre, or community pharmacy)?
        - In relation to [patient name]'s medicines, do you ever use a community pharmacy to get their medicines? (ask if community pharmacy not mentioned above)
            - How often do you use a community pharmacy to get [patient name]'s medicines? Do you use the same one?
- How do [patient name]'s sight problems affect how they take their medicines? (for example: reading medicines' labels, telling tablets apart? measuring liquid medicines? identifying expiry dates?) (Ask about different dosage forms: tablets, eyedrops, injections, creams)
        - *Prompt:* How do you help [patient name]'s to take their medicines?
- Has anyone ever checked to see that [patient name] is able to get or take their medicines? Have they offered any advice? What advice did they offer?

- What would be helpful to you in getting [patient name]'s medicines and getting [patient name] to take their medicines in the future?
    - *Prompts:* Talking insulin pens, different colour packages, braille on package, dispensing medicines in different bags, dispensing in original package not bags or vials

Social/professional role and identity

- What do you think your responsibility is/responsibilities are in relation to [patient name]'s medicines?
    - *Prompts*
        - Is there anything apart from these things that you should be responsible for?
        - Do other people have some responsibilities for [patient name]'s medicines? What are these responsibilities?

Beliefs about capabilities

- In what situations do you feel confident about [patient name] getting and taking their medicines?
- In what situations do you not feel confident about [patient name] getting and taking their medicines?
    - *Prompt:* What would help you to overcome these problems or difficulties?

Optimism

- How optimistic are you that you can overcome any problems with [patient name]'*s* medicines?
- What would make you feel less optimistic about overcoming any problems with [patient name]'s medicines?

Beliefs about consequences

- What do you think are the benefits of [patient name] taking their medicines as prescribed?
    - *Prompts:*
        - For the patient; caregiver; relatives

        - In general, what are the benefits of patients taking their medicines as prescribed?
        - Can you think of any short- and long-term consequences of [patient name] taking their medicines as prescribed?
- Can you think of any disadvantages to [patient name] taking their medicines as prescribed?
- Are the benefits of [patient name] taking all of their medicines worth the possible disadvantages?

<u>Reinforcement</u>

- What would encourage you to get [patient name] to take their medicines?
    - *Prompts*
        - Seeing them feeling better, family support
- What would discourage you from getting [patient name] to take their medicines?
    - *Prompts*
        - Too many medicines, side effects

<u>Intentions</u>

- Do you intend to get [patient name]'s medicines from the pharmacist and get them to take their medicines as prescribed?
- What would prevent you from getting [patient name]'s medicines from the pharmacist and getting them to take it as prescribed?
    - *Prompt:* Can you tell me why?

<u>Goals</u>

- To what extent is it a priority for **you** that [patient name] takes their medicines?
- When do you think it is less important that [patient name] takes their medicines?

<u>Memory, attention and decision processes</u>

- In this question I am interested to know how you remember to get [patient name] to take their medicines. Do you have any reminders, prompts or routines?
    - *Prompt:* What is your usual routine? Is there something that/someone who helps you to remember to get [patient name] to take their medicines?
- Are there any circumstances in which you might just forget or find it difficult to get [patient name] to take their medicines?

<u>Environmental context and resources</u>

- What things would help you to get [patient name] to take their medicines?
    - *Prompts*
        - Pillbox, reminders
- What things might prevent you from getting [patient name] to take their medicines?
    - *Prompts*
        - Home environment, being away from home, being busy

<u>Social influences</u>

- Who would influence your decisions to get [patient name] to take their medicines?
    - *Prompts*
        - Other family, friends, pharmacist, doctor, nurse, optometrist, other healthcare professional
- Can you tell me more about how they influence/help you?
    - *Prompts:*
        - Is there anything that the pharmacist/pharmacy does that makes it easier for you to get [patient name] to take their medicines?
        - Is there anything that your pharmacist/pharmacy does that makes it harder for you to get [patient name] to take their medicines?
        - What role, if any, does any other healthcare professional (e.g. doctor, nurse, optometrist) play in helping you to get [patient name] to take their medicines as prescribed?
- Are all healthcare professionals providing care for [patient name] aware of [patient name] sight problems?
    - *Prompts*
        - Doctors, nurses, pharmacists
        - If yes – how did they know? If no, why is that?
        - Does [patient name] tell people about their sight problem and ask for help?
        - Does [patient name] use a white cane?

Emotion

- How does [patient name] taking their medicines make you feel?
    - *Prompts*
        - For example, Does it make you feel happy/sad/anxious/stressed?
- How does that influence your decision to get [patient name] to take their medicines?

Behavioural regulation

- Do you have something in place that could help you check whether or not [patient name] has taken their medicines?
    - *Prompts*
        - For example, a calendar, pillbox
- Can you tell me more about how this works for you?

Future Planning

"The research team is interested in developing a plan to support and try to help patients with sight problems and their caregivers with medicines. It has been useful to hear about your experiences in more detail as this will help us to try and understand what patients and their caregivers are struggling with when it comes to medicines, and therefore what aspects we should target as part of our approach to improve the use of medicines in the future."

- If you were to think about the way in which [patient name] gets and takes their regular medicines, can you tell me:
    - What works well with that process/system?
    - What could be done differently that may make things better?
- If [patient name]'s pharmacist arranged to sit down with you and/or [patient name] and go through all the medicines that [patient name] take, how would you feel about this?
    - What would you like to see happen as a result?

- Is there anything else that a pharmacist could do to help you with you role in helping [patient name] use their medicines?

Closing the interview

"That brings us to the end of the interview.

Is there anything else about [patient name] medicines that we should have talked about, but didn't?

Do you have any additional comments you would like to make?

Thank you very much for making the time to speak with me today."

[Turn the digital recorder off]
