# Supplementary material for: Barriers and Facilitators to Medicines Use in Patients With Vision Impairment: A Theory‐Informed Qualitative Study of Patients and Caregivers
Source: Health Expect. 2025 Mar 21;28(2):e70234. doi: 10.1111/hex.70234 (PMC11926560; doi:10.1111/hex.70234)
Supplement: Supplementary file 3 — Supporting information. [file HEX-28-e70234-s001.pdf]

Table S1 Determinants (facilitators and barriers) of patients' behaviour in obtaining and taking medications

TDF Domain	Facilitators	Barriers	Illustrative quotes
**Knowledge**	Good knowledge of medicines indications, doses, administering times, side effects…etc.<br><br>Knowledge of consequences of taking/ not taking medicines<br><br>Knowledge of certain aspects of medicines (e.g. colour/shape) to help differentiate them<br><br>Lack of/incorrect knowledge of side effects<br><br>Prolonged use of medications<br><br>Patients' interest in knowing about their medications<br><br>Knowledge of Braille	Lack of/incorrect knowledge of side effects<br><br>Changing brands<br><br>Reading medicine leaflets inducing fear of side effects<br><br>Lack of/incorrect knowledge of medicines' names (names being in English was an additional barrier)<br><br>Illiteracy<br><br>Newly prescribed medications<br><br>Issues with knowing expiration dates<br><br>Limited information provided on medication labels	*"Vitamin D comes in pills that are somewhat like beads. I mean I will know them … The folic acid is yellow, I will just recognise it."* (PT01)<br><br>*"Well, I don't know about them* [side effects]. *I take them and know nothing."* (PT11)<br><br>*"* [I recognise them] *by the box […] I don't know English, you see. […] If it* [name] *was in Arabic, I'd know it."* (PT16)<br><br>*"…I spend one or two months and I get a different new box […] this is what messes things up for me."* (PT13)<br><br>*"By God, I took, I took maybe a year ago…I took three strips of an expired medication…without knowing."* (PT10)<br><br>*"...You'd find written 'Take one pill for 90 days.' Ok, a pill for 90 days, I don't know what this is. Why don't you tell me, well, after meal, before meal […] there are no instructions…"* (PT16)

Table S1 ***(cont'd)*** **Determinants (facilitators and barriers) of patients' behaviour in obtaining and taking medications**

TDF Domain	Facilitators	Barriers	Illustrative quotes
**Skills**	Having a method to recognise medicines (shapes/colours/touch/sound)<br><br>Using visual aids (e.g. magnifiers)<br><br>Memorising colours, shapes, dosing schedule<br><br>Relying on marks (lines) drawn on boxes to know how a medicine should be taken<br><br>Making certain marks (e.g. a dent on the cover, folding or tearing a small section of the box) to differentiate medicines<br><br>Ability to read information in Braille<br><br>Using smart phones as a magnifier or to set alarms<br><br>Using a pill organiser<br><br>Using available resources to obtain medications when they run out (courier company, community pharmacies)<br><br>Organisation skills<br><br>Long experience with using medications<br><br>Relying on sound for insulin administration	Inability to read numbers/labels/instructions<br><br>Inability to recognise different colours<br><br>Recognising medicines that look similar or dispensed in identical bottles<br><br>Lack of physical ability to administer certain medication formulations (e.g. eye ointments, eye drops, insulin pens)	*"It is basically obvious by its shape. And even when I…like when the light is off or something. I hold it and feel it. Xolamol®* [Dorzolamide and Timolol] *is a little short and wide. Iopidine®* [Apraclonidine]*, no, it is short and narrow."* (PT24)<br><br>*"I...I…by God I cannot see it well. I've put a mark on its cover. […] Like, I got a knife and made a cut across the cover itself."* (PT10)<br><br>*"The expiration date, I either get a magnifier or let someone read it for me." (PT23)*<br><br>*"No, no, I just take them myself, Thank God. I set the alarm on my mobile and just go with it."* (PT01)<br><br>*"Well, look, thank God I have the shapes memorised, have everything memorised."* (PT01)<br><br>*"I cannot put it. I mean you feel the medication got outside the eye. The medication is wasted. […] So, I have to have my brother put this ointment specifically inside the eye so that I don't run out."* (PT21)

Table S1 *(cont'd)* Determinants (facilitators and barriers) of patients' behaviour in obtaining and taking medications

TDF Domain	Facilitators	Barriers	Illustrative quotes
**SPRI**	Patients believing medication use is their responsibility	Beliefs about a visually impaired person not being able to be totally independent	*"I am the one who gets them, I am the one who takes them, […] I even know when they will run out. Before they run out, I must go to the hospital to get them."* (PT05)<br><br>*"…I rely on them* [family] *to help me, because as a blind person, I mean I cannot be totally independent if there is no Braille writing…"* (PT01)
**Beliefs about capabilities**	Beliefs that medication use is easy and is a routine process<br><br>Availability of medicines<br><br>Availability of resources (e.g. vision aids, Braille information, transportation)<br><br>Vision being at a certain level or vision being 'restored'	Unavailability and expense of medications<br><br>Certain medication dosage forms<br><br>Getting older<br><br>No information in Braille<br><br>Vision worsening<br><br>Being embarrassed about using medications at younger age	*"…It* [medicine] *has become my routine, part of my daily routine. I mean it is, like, ok. I don't feel it is something new or strange to me."* (PT22)<br><br>*"… I mean since I can see now…I mean maybe people with sight weaker than me are the ones who need them [drops] to have like a certain shape, a certain colour. But, I… for me I mean, thank God, it does not make a difference to me."* (PT24)
**Optimism**	Small number of medications<br><br>Religious beliefs (God will help)<br><br>Vision improving	Previous negative experiences with medication use<br><br>Difficulty in obtaining medications	*"I mean my mother carries three bags […] three bags of medications […] so, compared to her I am better…I have for blood pressure, for diabetes, simple things I mean."* (PT12)<br><br>*"Well, look, I tried…[…] I had to put* [the ointment] *on the tip of a finger and put it at the bottom of the eye, but…it does not work! It does not work! That's why I stopped it the three or four days…"* (PT30)

Table S1 *(cont'd)* Determinants (facilitators and barriers) of patients' behaviour in obtaining and taking medications

TDF Domain	Facilitators	Barriers	Illustrative quotes
**Beliefs about consequences**	Beliefs about benefits of medications to patient and family	Beliefs about negative consequences/side effects of medications<br><br>Beliefs about benefits being few/not always outweighing the disadvantages<br><br>Beliefs that taking more of a medication than prescribed leads to more improvement	*"No, I swear they are very um useful. Because if I don't take them, I get sick. The sugar goes up, the blood pressure goes up…"* (PT15)<br><br>*"The hospital medication…it has benefits and has harms, that's why I… I cannot confirm that the benefits are more."* (PT01)<br><br>*"Because, because people say it causes umm…in, in the ear, like you won't hear well… […] cholesterol [medicine] causes this in the ears so I thought: […] 'I've lost my sight. Should I lose my umm hearing too! Better get rid of it.'"* (PT10)
**Reinforcement**	Being healthy and well<br><br>Avoidance of complications<br><br>Maintaining life independence<br><br>Family encouragement<br><br>Doctor's directions to take medications	Side effects<br><br>Number of medicines<br><br>Negative emotions (e.g. feeling down, feeling bored of medication use)<br><br>Fear that medications may not be needed but prescribed because of the doctor's diligence	*"I mean I have moments of feeling bored of using [medications]. But, I'd go back and see that the benefit of using is more important than the boredom I'm feeling. So, it gives me a motivation to continue."* (PT30)
**Intentions**	Positive intentions to obtain and take medications	Unavailability of medicines<br><br>Getting bored of medication use	*"I don't risk it. I have to! I cannot leave it. If I leave it, I get sick."* (PT15)

Table S1 *(cont'd)* Determinants (facilitators and barriers) of patients' behaviour in obtaining and taking medications

TDF Domain	Facilitators	Barriers	Illustrative quotes
**Goals**	Goal of taking medications as prescribed<br><br>Clinical goals (e.g. maintaining health, avoidance of complications)<br><br>Priority for medications patients consider more important than others	Some medications considered less of a priority than others<br><br>Distractions (e.g. work, visitors)<br><br>Less priority given to medications when feeling ill	*"No, that's ok, I go out and take them with me to the same place, with me. I mean the most important thing to me is my health."* (PT22)<br><br>*"I left them once before. I mean my stomach caused me problems once before […] and I left them. I took them the next day."* (PT09)<br><br>*"…There is a medication which is Combagin* [sic] [Combigan®: Brimonidine and Timolol] *[…] you know when you feel it is an addiction […] even if he* [doctor] *told me to stop it, I may use it behind his back gradually…"* (PT21)
**MADP**	Paying attention to medications when out of normal context (e.g. on holidays, special occasions)<br><br>Paying attention to medications deemed to be of greater importance<br><br>Memorising medication use instructions, shapes<br><br>Alarm on mobile phone<br><br>Family support<br><br>Having a routine<br><br>Organising medicines in a certain way	Reduced attention to medications when out of normal context (e.g. on holidays, special occasions)<br><br>Less attention paid to medications deemed to be of lesser importance<br><br>Forgetting to take medications<br><br>Night-time medications<br><br>Relying on memory	*"I have an alarm for every medicine with the medicine name. I mean, when the alarm goes off, the name of the medicine appears on the screen…"* (PT01)<br><br>*"I've set them up. The one for the gland I've set it up, pray at dawn [and] take it. […] and the um injections, you know, before breakfast, before lunch, before dinner. I've just organised my time."* (PT28)<br><br>*"The one for fats, well, to tell you the truth, I do not take it. I forget it. It is at bedtime, and I am kind of … forgetful."* (PT10)<br><br>*"I've put medications at her* [daughter] *house and at her sister's house. […] So that I won't forget. I am afraid to forget to bring medications with me."* (PT04)

Table S1 ***(cont'd)*** **Determinants (facilitators and barriers) of patients' behaviour in obtaining and taking medications**

TDF Domain	Facilitators	Barriers	Illustrative quotes
**ECR**	Access to medicines (medications being available and easily obtained)<br><br>Availability of alternative medicine brands<br><br>Easy process for ordering medications from the pharmacy (e.g. pharmacy website, courier services)<br><br>Availability of transport to get to the pharmacy<br><br>Braille information<br><br>Smart phone (alarm/speaker/screen reader)<br><br>Pill organisers<br><br>Glasses, magnifiers<br><br>Different colour/shape of medication packs or packs with differentiating marks<br><br>Specific room/cupboard/box/bag<br><br>Keeping medicines close by<br><br>Being fast-tracked at the pharmacy<br><br>Identity card that indicates disability which helps patients bypass pharmacy queues	Difficulties in access to medicines (e.g. in small cities, medications being dispensed only from central pharmacies)<br><br>Lack of/unclear Braille information on medication packs<br><br>Small font medication instructions<br><br>Changing brands by pharmacy<br><br>Queuing system at the pharmacy<br><br>Pharmacy layout<br><br>Suspension of medication delivery service<br><br>Difficulty in obtaining doctor appointments<br><br>Pill organiser (concerns about medications getting contaminated, absence of Braille information)<br><br>Expense of visual aids<br><br>Requirement of a prescription by community pharmacies before dispensing medications<br><br>Patients not using a white cane which contributes to making them less identifiable	*"The expiration date, I either make my brother, like, read it for me, or get the mobile phone and enlarge with it."* (PT21)<br><br>*"They [family] come and make it [pill organiser] for me and prepare everything for me in this bag and put it aside in the drawer. This drawer is beside my bed."* (PT28)<br><br>*"When I went to* [Name of hospital]*, they have a nice service there. They have assigned staff to help the visually impaired, guide them to the clinics…"* (PT23)<br><br>*"Some medications do not come here to us in villages […] So, it is difficult for you to find it. You need to go to the big city…"* (PT12)<br><br>*"There are many doors* [means windows] *at the pharmacy, maybe seven or eight. Umm those who bring the medication, I don't know where they are calling me from…that is what I mean."* (PT10)<br><br>*"…Sometimes the number would come up, I cannot see the screen. I cannot see it to read or anything." (PT20)*<br><br>*"I have a speaker, screen reader […] in the iPhone."* (PT01)

Table S1 *(cont'd)* Determinants (facilitators and barriers) of patients' behaviour in obtaining and taking medications

TDF Domain	Facilitators	Barriers	Illustrative quotes
**Social influences**	Social support from physicians<br><br>Social support from pharmacists<br><br>Social support from caregiver and other family members<br><br>Social support from other patients at the pharmacy to navigate queuing system	Assuming all HCPs know about patient's VI<br><br>Not disclosing VI to HCPs if not their speciality<br><br>Limited information provided by pharmacists<br><br>Beliefs about pharmacist role (Doctor makes changes and pharmacist gives medicines according to orders)<br><br>Difficulty in discussing medication issues with hospital pharmacists<br><br>Improper advice/help from people around patient	*"…The pharmacist now may know better than the doctor […] I sat once with a doctor who had someone from the pharmacy with him. While writing [the prescription] he said 'Ok, do you have this mediation? Does it work for him or should we give him more?' […] I was happy! […] I thought 'Everyone* [doctor] *should have one* [pharmacist] *with him…'"* (PT16)<br><br>*"I need the pharmacist only for dispensing medication. But, for the instruction process or the essentials, the treating doctor."* (PT30)<br><br>*"…If you're not like careful! Careful to discuss, otherwise, the pharmacist won't …won't deal with you."* (PT01)<br><br>*"He* [pharmacist] *knows* [about VI] *of course. Because of the medications I take from him and so on."* (PT29)<br><br>*"It* [knowing about VI] *is not his business, he* [pharmacist] *just dispenses the medication and that is it."* (PT20)<br><br>*"Yeah, but why I don't ask the pharmacist at the hospital? Because […] there would be many people waiting and there are people who cannot… older people, cannot bear it, you know?"* (PT22)

Table S1 ***(cont'd)*** **Determinants (facilitators and barriers) of patients' behaviour in obtaining and taking medications**

TDF Domain	Facilitators	Barriers	Illustrative quotes
**Emotion**	Feeling comfortable with medication use<br><br>Feeling compelled to use medications<br><br>Anxiety about making errors with medication use encourages patients to seek support<br><br>Anxiety about having complications encourages patients to take medications as prescribed	Fear caused by reading medicines side effects in leaflets<br><br>Anxiety associated with high number of medications<br><br>Feeling down<br><br>Feeling embarrassed by using medication around people	*"I mean because of the many medications, I get annoyed with them, get upset. Because they are too many. Since I was young, and I'll use it, like, forever."* (PT23)<br><br>*"I used to be bothered by people's looks, and I did not like taking my drop in front of anyone even our relatives…"* (PT24)
**Behavioural regulation**	Memory<br><br>Planning administration times<br><br>Alarm on mobile phone<br><br>Counting medicines/pills<br><br>Checking pill organisers<br><br>Recording medication use (e.g. in a journal)<br><br>Family support<br><br>Clinical outcomes	Lack of method to monitor medication use<br><br>Being used to medications leading to complacency	*"I just count the medicines I have taken."* (PT01)<br><br>*"…So, I take breakfast and go take the medicines and that's it. And when I have dinner at night, around sunset, I take the rest."* (PT05)<br><br>*"…If I'm doubtful…doubtful about not taking something […] They [family] would be around, [I'd ask] "Did I take this medication or not?"…they'd tell me."* (PT10)<br><br>*"…So, I take breakfast and go take the medicines and that's it. And when I have dinner at night, around sunset, I take the rest."* (PT05)

Abbreviations: ECR= Environmental context and resources; MADP= Memory, attention, and decision processes; PT= Patient; SPRI= Social/professional role and identity

Table S2 Determinants (facilitators and barriers) of caregivers' behaviour in assisting visually impaired patients with obtaining and taking medications

TDF Domain	Facilitators	Barriers	Illustrative quotes
**Knowledge**	Good knowledge of medicines indications, doses, administering times, side effects…etc.<br><br>Knowledge of consequences of taking/not taking medicines<br><br>Lack of/incorrect knowledge of side effects<br><br>Caregiver's interest in knowing about medications	Knowledge of side effects<br><br>Changing brands<br><br>Lack of/incorrect knowledge of medicines' names<br><br>Medication names being in English<br><br>Newly prescribed medications<br><br>Lack of medication information provided by the pharmacist	*"I know that every medication has a side effect, but, like, it has an advantage. I know this."* (CG10)<br><br>*"…Yeah, of course we want, like, to get more information."* (CG03)<br><br>*"No, no, no, he* [pharmacist] *does not have…there are no information whatsoever, sister. […] You are a patient, take your medications and go."* (CG09)
**Skills**	Teaching the patient about medicines<br><br>Monitoring outcomes<br><br>Creating identifying marks to help patient recognise medicines<br><br>Using a pill organiser<br><br>Using available resources to obtain medications when they run out (Smart phone application for ordering medications from hospital pharmacy, buying medicines from community pharmacies)<br><br>Organisation skills (particularly when caring for more than one patient)	None identified	*"I mean, we taught her, this green box is aspirin. Yeah, and the [one for] diabetes we taught her that this big one she has to break it, one with breakfast and one with dinner."* (CG03)<br><br>*"I start arranging it* [pill organiser] *for them on Saturday night because it starts from Saturday to Friday. I arrange it, fix it, and give it to them."* (CG01)<br><br>*"Yeah, um the hospital made an application. In the case medicines run low, I put the type of medication, write it down and order it. […] either I go pick it up or order delivery and that would have a charge."* (CG11)

Table S2 *(cont'd)* Determinants (facilitators and barriers) of caregivers' behaviour in assisting visually impaired patients with obtaining and taking medications

TDF Domain	Facilitators	Barriers	Illustrative quotes
**SPRI**	Caregivers believing that it is their role to help patients with a range of medication use aspects<br><br>Beliefs that patients should be more responsible for their medication use	Patient's prolonged use of medications	*"We are the ones who give it to her. […] We give it to her in her hand and my mother puts it in her mouth."* (CG09)<br><br>*"This is my mother! I should be helping my mother. Who would help her other than me!"* (CG02)<br><br>*"…I'd like him to be attentive, I mean. Attentive on his own, I mean. […] I'd tell him 'Put a reminder for yourself on the mobile phone if you, like, have a drop' […] I mean 'I will not be there for you forever, your father will not be there for you forever. Depend on yourself!'"* (CG07)
**Beliefs about capabilities**	Confidence in patient's ability to manage certain aspects of medication use<br><br>Hopes that a young patient's ability to manage medications increases with age<br><br>Vision being 'restored'	Lack of confidence in patient's ability to manage aspects of medication use<br><br>Patient's reluctance to take medication<br><br>Patient's illiteracy	*"There are people who are old, but they understand. They understand. They take care of themselves, take care of their medications […]. But my mother, no. There should be someone around to give her the medication."* (CG09)<br><br>*"My mother is illiterate. […] That is what she can do, like 'Put it in the box and I'll take it.'"* (CG04)<br><br>*"…Maybe four medicines, all of them were syrups. These, I had to be careful that I give it to them myself. […] Because, you know, it's hard to draw the milli* [millilitres] *and so on. They can't see."* (CG01)

Table S2 ***(cont'd)*** **Determinants (facilitators and barriers) of caregivers' behaviour in assisting visually impaired patients with obtaining and taking medications**

TDF Domain	Facilitators	Barriers	Illustrative quotes
**Beliefs about capabilities *(cont'd)***			*"I always tell him, like, 'Use your phone, put an alarm on your phone. At least it'd remind you that you have medications! Just put it on your own, you know how. […] But, he does not want to!"* (CG07)
**Optimism**	Patient's ability to manage medication use<br><br>Creating a new method for organising medications<br><br>Patient's vision being 'restored'<br><br>Patients learning skills that can help them manage their own medications	Patient's reluctance to take medication<br><br>Patient's worsening vision	*"Well, I'm hoping for the best. Because…because I'm making this new organisation…"* (CG10)<br><br>*"He can put them, but he does not accept them. I mean, he does not want to, you see? Something, like, something psychological."* (CG07)<br><br>*"Yeah, if his sight returns or if for example there was education […] someone teaching him how to read, how to tell things apart…"* (CG06)
**Beliefs about consequences**	Beliefs about benefits of medications to patient and family	Beliefs about negative consequences/side effects of medications	*"Well, we don't go out, or come or go anywhere, or travel or anything. In our home. Hospital [to] home. Home [to] hospital."* (CG01)
**Reinforcement**	Patient being healthy and well<br><br>Avoidance of complications<br><br>Avoidance of patient's hospitalisation<br><br>Obligation to support parents<br><br>Reducing the emotional burden (e.g. feeling tired) on the caregiver	Patient's reluctance to take medication<br><br>Negative consequences/side effects of medications<br><br>Number of medicines<br><br>Beliefs about medications being ineffective	*"Well, um the first thing is doing good to my mother."* (CG10)<br><br>*"The tiredness…the tiredness, the tiredness. By God, I am honestly tired! I've got diabetes, took psychiatric medication. By God, I am tired!"* (CG01)

Table S2 ***(cont'd)*** **Determinants (facilitators and barriers) of caregivers' behaviour in assisting visually impaired patients with obtaining and taking medications**

TDF Domain	Facilitators	Barriers	Illustrative quotes
**Intentions**	Positive intentions to help patients obtain and take medications<br><br>Behaviour being a routine	Limited income<br><br>Difficulty with hospital appointments (e.g. travelling for the appointment)<br><br>Forgetting to obtain medications<br><br>Unavailability of medicines	*"…Some medications, you see, are 300 or 400* [Riyals] *[…] the income and the, the, distance. We are in* [Name of major city in southern KSA] *and have to go to Riyadh."* (CG09)
**Goals**	Goal of helping patient take medications as prescribed<br><br>Clinical goals (e.g. maintaining health, avoidance of complications)	Patient's reluctance to take medication<br><br>Distractions (e.g. work, visitors)<br><br>Less priority for medications patient/caregiver considers less important/beneficial than others	*"I miss work, like an hour or two, to get my mother's medication and come back."* (CG10)<br><br>*"He* [patient] *doesn't like to put it. So, it is extremely difficult to put it for him. It is even with a fight sometimes. Because it is essential, I mean."* (CG07)<br><br>*"Less important because I feel they* [eyedrops] *are not useful […] Every time I ask him* [husband] *'Do you feel there is improvement?', he'd say 'No, it is the same.' And I feel he is the same. He is the same. There is no change!"* (CG06)
**MADP**	Paying attention to medications when out of normal context (e.g. being absent, special occasions)<br><br>Avoidance of patients having complications/ hospitalisation<br><br>Support at home (e.g. housekeeper, other family members)	Reduced attention to medications when out of normal context (e.g. travelling, death in family)	*"…She only goes out with me. And when she does, I take her medications with me." (CG02)*<br><br>*"I mean I don't set a mobile phone* [alarm] *or set anything, no, I don't…I already got used to them."* (CG07)

Table S2 *(cont'd)* Determinants (facilitators and barriers) of caregivers' behaviour in assisting visually impaired patients with obtaining and taking medications

TDF Domain	Facilitators	Barriers	Illustrative quotes
**MADP *(cont'd)***	Having a routine and getting used to helping patient with medications<br><br>Using alarms on mobile phone<br><br>Keeping medications in sight		*"…It would be with me in the bag. I mean always with me. I put it beside me. This way I can remember it."* (CG06)<br><br>*"I swear I'd wake up from deep sleep and umm give them the medication. […] Because I'm tired, really tired! I suffered a lot with this, medications, and the hospital admission."* (CG01)
**ECR**	Medications being available and easily obtained<br><br>Bigger font for medication instructions<br><br>Differentiating shapes/marks, Braille info<br><br>Pill organiser (useful if it was also coloured)<br><br>Organising boxes/bags made by the caregiver<br><br>Storing medications close to the patient and at the same place<br><br>Pharmacy services (e.g. ordering refills through a smart phone application or phone calls)	Unavailability of medicines<br><br>Difficulty in obtaining medicines from the pharmacy (long process, limited amount dispensed)<br><br>Limited income<br>Difficulty with hospital appointments<br><br>Concerns that using a pill organiser will lead to medications being contaminated, reducing efficacy or lead to errors<br><br>Similar looking medication packs leading to confusion	*"But the best thing to guarantee she has taken the medication is this divider [pill organiser]."* (CG01)<br><br>*"…Before the App [sic], I used to go to the pharmacy and give them a paper where I write all the needed medications […] and leave the paper there and go. After 24 hours, I'd go back again. They either approve it or don't approve it."* (CG11)<br><br>*"Like, I'd come to the pharmacist today and he'd say 'Well, the blood pressure medication is not available, come tomorrow. And the Aspirin is not available, come…come on…x day.' So, I would have to excuse myself from work in a month two to…to four times sometimes!"* (CG10)<br><br>*"…No, no, no, we don't bring this [pill organiser] because…because this may make one make errors […] it could also get contaminated."* (CG02)

Table S2 *(cont'd)* Determinants (facilitators and barriers) of caregivers' behaviour in assisting visually impaired patients with obtaining and taking medications

TDF Domain	Facilitators	Barriers	Illustrative quotes
**Social influences**	Social support from physicians<br><br>Social support from pharmacists<br><br>Social support from other family members/ housekeeper	Assuming all HCPs know about patient's VI<br><br>Patient's reluctance to take medication<br><br>Patients insisting on taking medications in a way that is different from what was prescribed<br><br>Difficulty in discussing medication issues with community pharmacists<br><br>Difficulties with dealing with pharmacists<br><br>Lack of enough support from pharmacists	*"I talked to the doctor who communicated with her at the pharmacy, her name is* [Name of pharmacist], *and he said, 'Give her your number and contact her on Whats* [App], *anything that is difficult for you''. Yeah, I take a photo and tell her 'This I don't understand, explain it to me…'"* (CG01)<br><br>*"The pharmacist of course knows [of the VI] when he sees the medications."* (CG05)<br><br>*"…He* [patient] *is not accepting* [them]. *He is irritated I mean, very irritated by them. With a fight, I swear it is with a fight I mean …for him to put the thing."* (CG07)<br><br>*"Well, I don't remember that a pharmacist sat and told us anything. […] I mean I'd get the medication and he'd tell me that the instructions are on the paper."* (CG11)<br><br>*"The doctor decides. He is the one who increases the dose. He is the one who reduces the dose. It is up to the doctor."* (CG02)<br><br>*"No, no, no, he [pharmacist] cannot. They are busy with other people."* (CG09)

Table S2 *(cont'd)* Determinants (facilitators and barriers) of caregivers' behaviour in assisting visually impaired patients with obtaining and taking medications

TDF Domain	Facilitators	Barriers	Illustrative quotes
**Emotion**	Positive emotions associated with patient taking medications<br><br>Anxiety about patient's need for hospital admission leading to caregiver being more careful about medications	Negative emotions caused by people around the caregiver	*"They'd* [relatives] *say 'You should not insist on going and getting* [medicines] *[…] He is blind and will remain blind. If he does not take them today, he can take them tomorrow. It won't affect him.' So, I feel broken and be sensitive about it. This is what hurts me…"* (CG06)<br><br>*"I swear I'd wake up from deep sleep and umm give them the medication. […] Because I'm tired, really tired! I suffered a lot with this, medications, and the hospital admission."* (CG01)
**Behavioural regulation**	Asking/calling the patient<br><br>Planning administration times<br><br>Checking medicines/pill organisers<br><br>Watching the patient take the medications<br><br>Symptoms/laboratory<br><br>Housekeeper helping patient	Lack of method to monitor patient's medication use<br><br>Being used to medications	*"She is the one who takes them and I'm standing watching."* (CG02)<br><br>*"…And I tell them that I know if you took the medicine. Lab tests tell me everything!"* (CG01)<br><br>*"I'd call her at half past six and say, 'Go take the medicine' and she takes her medication. The housekeeper would be with her. […] I'd call the housekeeper and ask her, she'd say 'Yeah, she took the medication.'"* (CG01)

Abbreviations: CG= Caregiver; ECR= Environmental context and resources; MADP= Memory, attention, and decision processes; SPRI= Social/professional role and identity
